# Protein Analysis of Pollen Tubes after the Treatments of Membrane Trafficking Inhibitors Gains Insights on Molecular Mechanism Underlying Pollen Tube Polar Growth

**DOI:** 10.1007/s10930-021-09972-x

**Published:** 2021-03-09

**Authors:** Monica Scali, Alessandra Moscatelli, Luca Bini, Elisabetta Onelli, Rita Vignani, Wei Wang

**Affiliations:** 1 Department of Life Sciences, University of Siena, Siena, Italy; 2 Department of Biosciences, University of Milano, Milano, Italy; 3 College of Life Sciences, Henan Agricultural University, Zhengzhou, China

**Keywords:** *Nicotiana tabacum* (L.), Pollen tube, Proteome, Membrane Trafficking Inhibitors, Differentially Abundant Proteins (DAPs)

## Abstract

Pollen tube elongation is characterized by a highly-polarized tip growth process dependent on an efficient vesicular transport system and largely mobilized by actin cytoskeleton. Pollen tubes are an ideal model system to study exocytosis, endocytosis, membrane recycling, and signaling network coordinating cellular processes, structural organization and vesicular trafficking activities required for tip growth. Proteomic analysis was applied to identify *Nicotiana tabacum* Differentially Abundant Proteins (DAPs) after in vitro pollen tube treatment with membrane trafficking inhibitors Brefeldin A, Ikarugamycin and Wortmannin. Among roughly 360 proteins separated in two-dimensional gel electrophoresis, a total of 40 spots visibly changing between treated and control samples were identified by MALDI-TOF MS and LC–ESI–MS/MS analysis. The identified proteins were classified according to biological processes, and most proteins were related to pollen tube energy metabolism, including ammino acid synthesis and lipid metabolism, structural features of pollen tube growth as well modification and actin cytoskeleton organization, stress response, and protein degradation. In-depth analysis of proteins corresponding to energy-related pathways revealed the male gametophyte to be a reliable model of energy reservoir and dynamics.

**Supplementary Information:**

The online version contains supplementary material available at 10.1007/s10930-021-09972-x.

## Introduction

Pollen germination and pollen tube growth are essential processes in controlling sexual plant reproduction. Integrating many internal and external signals, these processes are highly sensitive to environmental fluctuations such as temperature, light, and drought [7]. Therefore, pollen functionality is a major focus in applied research aimed at improving plant productivity. Besides its importance for sexual reproduction, the pollen tube is a valid model for studying endocytosis and membrane recycling, both involved in tip growth [11].

The pollen tube is characterized by fast and energy-consuming polarized cell expansion called tip growth, depending on a highly efficient vesicular transport system largely mobilized by the actin cytoskeleton [35, 54]. The actin organization has been extensively studied in vivo, showing distinct arrays in the tip and in the subapical regions [10]. In particular, the apical fringe requires actin filaments nucleation by formin AFH5, which is localized in the PM apical flanks [16]. The rapid fringe dynamics is further regulated by a plethora of actin-binding proteins and it has been shown that BFA treatments affects membrane trafficking and destroys the actin fringe [32, 60].

By actin-dependent cytoplasmic streaming, Golgi-derived secretory vesicles provide new materials for the plasma membrane, remodel the composition of the cell wall (carrying mostly pectins) and deliver a wide variety of proteins to the pollen tube surface, including signaling molecules and hydrolytic enzymes that could be important for pollen tube growth through the stylar transmitting tissue [76]. The intense cytoplasmic streaming and membrane trafficking require high ATP consuming. It was stated that mitochondria respiration represents the main mechanism for energy creation and the accumulation of mitochondria behind the clear zone supports the need of local ATP production for exo-endocytosis, actin cytoskeleton dynamics and PM localized ATPase functionality [17, 53, 63]. In addition, fermentation and plastidial glycolysis were also hypothesized as alternative mechanisms for energy production in low oxygen concentration state [49, 59].

Membrane quantity delivered by exocytosis to the cell apex exceeds that required for tip extension, suggesting the presence of a pathway based on endocytosis for active membrane recovery existing in the tip [31, 43]. The apical accumulation of secreted esterified pectins and pectin methylesterases I (PMEIs) in the apical cell wall of *Nicotiana tabacum* pollen tubes [56] suggests that exocytosis occurs at the pollen tube apex and that the excess material needs to be recycled via lateral endocytosis [19]. Further experimental evidence is in agreement with this model. Live-cell fluorescence microscopy analysis performed using GFP fused to the pollen specific *Nicotiana tabacum* PME shows an exclusive pollen tube apical cell wall labelling [75]. Further, the immunofluorescence analysis of the clathrin light chain (CLC) in *Nicotiana tabacum* pollen tubes results in a clear labelling of the lateral plasma membrane (PM) [23]. The presence of lateral endocytosis was also confirmed by Moscatelli et al. [44]. Lateral positively charged nanogold endocytosed at the pollen tube PM is mostly sent to the Golgi apparatus and reused in the secretory endomembrane system and partly transported to the vacuole for degradation through the trans Golgi network (TGN).

In the same fashion, inhibition of actin polymerization by Latrunculin B shows that PM internalized in the shank is delivered to prevacuolar compartments (PVCs) and then to vacuoles in an actin filaments (AF)-dependent way [46]. An alternative model of tip growth proposes the presence of apical endocytosis capable of compensating lateral secretions. Recent evidence supports that the PM internalized in the apex mostly goes to the degradation pathway bypassing the Golgi/TGN apparatus [44] in a MT-dependent manner [33].

Endocytosis seems to be involved also in the maintenance of apical polarity, a fundamental process for pollen tube growth guidance. Rapid tip growth requires a dynamic spatiotemporal control of the polar domains as well as a dynamic coordination between the apical and the lateral domains. Li et al. [39] proposed a novel model for the polar establishment and maintenance in rapidly growing pollen tubes. They identified a ROP1 enhancer4 protein (REN4) able to act in the spatiotemporal coordination between the apical and lateral domains, by integrating the apical ROP1 GTPase signaling system with the collar/apical clathrin mediated endocytosis (CME) pathway. More in detail, the apical active ROP1 seem to be able to interact with the lateral REN4 protein, inducing their CME-dependent removal from the PM and generating a dynamic demarcation that separates and coordinates the apical and lateral cortical domains, a very important process for the stabilization of pollen tube growth direction.

In the light of these studies, the presence of endocytosis processes both at the apex of growing pollen tubes and in the lateral regions [27], relies on a well organised vesicles trafficking supported by the activity of a dynamic cytoskeleton. An accurate signaling network is involved in regulating cellular processes required for tip growth, however the details of how structural organization and vesicular trafficking activities are coordinated are still partially revealed [29]. Biochemical approaches may provide an accurate and deep understanding of the molecular processes involved in the regulation of membranes trafficking and of how several signaling pathways can interact generating a signaling network able to modulate the rapid growth of pollen tubes. High resolution 2-DE, together with mass spectrometry, are valid tools to determine the protein composition of the various endomembrane compartments, monitoring the vesicles trafficking [28].

In the present study, the general effect of membrane trafficking inhibitors as Ikarugamycin (IKA), Brefeldin A (BFA), and Wortmannin (WMN), which inhibit clathrin-dependent endocytosis, ER-Golgi and vacuolar membrane sorting respectively, was investigated on *Nicotiana tabacum* pollen tubes. Although targets of these inhibitors have been characterized as intrinsic membrane proteins, the effects on the expression of membrane associated or soluble proteins have not investigated.

The experimental approach comprises two-dimensional electrophoresis (2-DE) and Matrix Assisted Laser Desorption/Ionization–Time-Of-Flight Mass Spectrometry (MALDI-TOF MS) and Liquid Chromatography Electrospray Ionization Tandem Mass Spectrometric (LC–ESI–MS/MS), with the aim of identifying proteins whose abundance is affected by pharmacological analysis.

The results are expected to clarify the nature and the regulation of crucial molecular mechanisms for the maintenance of the secretory pathway in the pollen tubes, which is critical for biosynthetic trafficking to the PM and vacuoles. The results revealed that membrane trafficking inhibitors, although acting on specific targets, have pleiotropic effects on the pollen tube proteome.

## Materials and Methods

### Pollen Culture and Endocytosis Inhibitor Treatments

*Nicotiana tabacum* (L.) pollen was collected from plants in the Botanical Garden of Siena University, dehydrated by incubation for 12 h in a box containing silica gel, and then stored at –20 °C. Before germination 100 mg of pollen was hydrated in a humid chamber overnight. Pollen (2.5 mg/mL) was germinated in BK medium [9] containing 12% sucrose at 23 ± 2° C, and membrane trafficking inhibitors were added directly to the culture medium after 60 min. Stock solutions of BFA (3.5 mM), IKA (10 mM) and WMN (3 mM) were prepared in 0,2% DMSO and then used at a final concentration of 10 μM BFA, 5 μM IKA and 30 μM WMN. Different incubation times were tested for each inhibitor in order to estimate the best experimental conditions affecting the pollen proteome without altering the germination percentage and without stopping pollen tube growth. The best exposure times were evaluated in 90 min BFA, 100 min IKA and 60 min WMN. Furthermore, as controls, pollen tubes were grown both in BK medium containing 12% sucrose, and in BK medium added of 12% sucrose and 0.2% (v/v) DMSO, in agreement with the DMSO concentration used in inhibitors stock solutions; based on previous studies a DMSO concentration below 0.5% should not alter the normal growth of pollen tubes [20]. Control pollen tubes were observed by optical microscope using a 10 × 10 and 10 × 40 objectives. The lengths of control pollen tubes were calculated by ImageJ software (National Institutes of Health) [64].

### Pollen Tube Extracts

Pollen tubes were homogenized in two volumes of PEM buffer (100 mM Pipes pH 6.8, 5 mM EGTA, 1 mM MgCl2, 1 mM DTT, 1 mM PMSF, 10 mg/mL TAME, 10 mg/mL leupeptin, 10 mg/mL pepstatin A, 4 mM aprotinin, 8 mM antipain). After an initial centrifugation at 15,000 rpm for 30 min, the supernatant was further centrifuged at 40.000 rpm for 100 min to get the high-speed supernatant (HSS). The HSS was processed using the “phenol based” method reported by Wang et al. [77] with some improvements: the HSS was mixed with equal volume of Tris-buffered phenol (pH 8.0, Sigma) by shaking for 30 min, the mixture was centrifuged at 16,000 g for 15 min and the phenol phase was recovered; protein in the phenol phase was precipitated with five volumes of 0.1 M ammonium acetate in methanol, and centrifuged at 16,000 g for 10 min; the pellet was suspended in a solution of 10% trichloroacetic acid in acetone containing 0.007% DTT and incubated at -20 °C for 2 h; after centrifugation at 16,000 g for 10 min, the protein pellets were rinsed with five volumes of 80% ice-cold acetone containing 0.007% DTT at -20 °C overnight; after centrifugation, the pellet was washed with ice-cold acetone twice, air dried and solubilized in rehydration buffer (7 M urea, 2 M thiourea, 4% CHAPS, 20 mM DTT and 1% IPG buffer). The pellet was assayed for protein concentration by the Bradford protein assay [8].

### Two-Dimensional Electrophoresis (2-DE) and Image Analysis

Proteins (400 µg) were dissolved in 200 µL of rehydration buffer supplemented with 2% 4–7 IPG buffer and loaded onto 11 cm linear pH 4–7 strips (Bio-Rad, USA) by overnight passive rehydration at room temperature. The pH range 4–7 was used according to the study of Schwartz et al. [65], showing that in prokaryotes and eukaryotes, cytoplasmic proteins are more acidic and have an average pI value of about 5–6. IEF was carried on with the Ettan III system (BIO-RAD) with the following parameters: 250 V for 4 h, 1000 V for 2 h, increasing to 8000 V over 3 h and holding at 8000 V until 96 kVh. Focused strips were equilibrated in Buffer I (0.5 M Tris–HCl pH 6.8, 6 M urea, 30% glycerol, 2% SDS and 2% DTE) for 15 min followed for 15 min in Buffer II (composition the same as Buffer I but containing 2% idoacetamide instead of DTE) at room temperature. Afterward, SDS-PAGE was run in 12% Criterion XT Bis–Tris Precast Gel (Bio-Rad) at 180 V constant, then stained with Bio-Safe Coomassie (Bio-Rad) for 3 h and destained in double-distilled water until a clear background was obtained.

### PDQuest Analysis

Gel images were captured using the Fluor-S Multi-Imager (BIO-RAD) and analyzed by PDQuest Advanced 8.0.1 2D Gel Analysis Software (version 7.0, BioRad, Hercules, CA, USA Bio-Rad). 2D gel profiles were screened for DAPs identification. The images were cropped to the same size and shape, and spots were detected and matched automatically to a master gel selected by the software. The analysis comprised spot detection, gel matching and statistical analysis. Spot detection and matching were edited manually. The separate analysis of each sample included alignment of each treated gels to its reference image (control gels). Quantitative analyses were carried out after normalizing spot volumes in all gels to compensate for abundance-related variations. Selection of DAP spots was based on fold change abundance > 2.0, with a consistent change in the three technical replicas of the two biological extracts.

### Mass Spectrometry and Protein Identification

Protein identification was performed as described above. Spots of interest were manually excised, destained in ammonium bicarbonate 2.5 mM and acetonitrile 50% (v/v), and acetonitrile dehydrated. 2D gel-resolved proteins were rehydrated in trypsin solution (Sigma Aldrich, Italy) and in-gel protein digestion was performed by an overnight incubation at 37 °C. For MALDI-TOF MS, 0.75 mL of each protein digest was directly spotted onto the MALDI target and air-dried. 0.75 mL of an alpha-cyano-4-hydroxycynnamic acid matrix solution was added to the dried samples and allowed to dry again. Mass spectra were acquired using an Ultraflex III MALDI-TOF/TOF mass spectrometer (Bruker Daltonics, Billerica, MA, United States). Spectra were analyzed by Flex Analysis software v.3.0. Peptide mass fingerprinting (PMF) database searching was carried out in NCBInr or Swiss-Prot/TrEMBL databases set for *Viridiplantae* (Green Plants) using Mascot (Matrix Science Ltd., London, UK, http://www.matrixscience. com) on-line-available software with the following settings: experimental and theoretical PMF patterns with a D mass less than 100 ppm, trypsin as the digestion enzyme, one allowed missed cleavage, carbamidomethylation of cysteine as fixed modification, oxidation of methionine as variable modification. The parameters used to accept identifications were number of matched peptides, extent of sequence coverage, and probabilistic score.

Peptide digests not unambiguously identified were further analyzed performing peptide sequencing by tandem mass spectrometry. MS/MS analysis was performed on the Ultraflex III MALDI-TOF/TOF instrument and on a nanoscale LC–ESI–MS/MS system using a Micro- HPLC Pump Phoenix 40 (Thermo Finnigan, San Jose, CA) and a LCQ DECA IT mass spectrometer (Thermo) [5]. By the MASCOT MS/MS ion search software available online, MS/MS database searching was carried out in Swiss-Prot/TrEMBL database applying the following parameters: trypsin specificity, one missed cleavage allowed, peptide precursor mass tolerance: 100 ppm, fragment mass tolerance: 1.2 Da, peptide precursor charge state: + 2, carbamidomethylation of cysteine as fixed modification, oxidation of methionine as possible modification, and taxonomy: Viridiplantae (Green Plants).

For LC–ESI–MS/MS analysis, peptides were extracted in 50% (v/v) acetonitrile and 0.1% (v/v) TFA and concentrated by Speed-Vac (SC110A Savant Speed-Vac, Thermo), then MS/MS peptide sequencing was performed as previously described [5, 41]. MS/MS database searching was carried out in Swiss-Prot/TrEMBL database using Mascot MS/MS ion search software. The taxonomy was limited to *Viridiplantae* (Green Plants), peptide precursor charge was set to + 2 or + 3, and precursor and fragment peptides’ mass tolerances were 61.2 Da and 60.6 Da, respectively. Trypsin was selected as the digestion enzyme with one allowed missed cleavage. Carbamidomethylation of cysteine was assumed as fixed modification, while oxidation of methionine as possible one. Only peptides with individual ion scores p < 0.05 were significant.

## Results and Discussion

### Membrane Trafficking Inhibitors Alter the Expression of Soluble Proteome

**Fig. 1:**
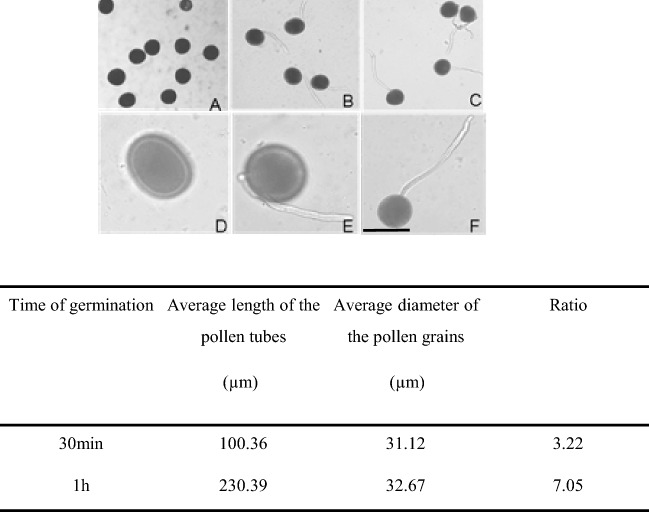
Pollen tube germination without membrane trafficking inhibitors. **a**, **d**, mature pollen grains; **b**, **e**, germinated pollen, 30 min; **c**, **f**, germinated pollen, 1 h. (**a**, **b**, **c** under 10 × 10; **d**, **e**, **f** under 10 × 40) **a**–**f** bar 50 µm

To investigate the protein changes induced by membrane trafficking inhibitors, *Nicotiana tabacum* pollen was allowed to germinate for 60 min, and then exo- and endocytosis inhibitors were added directly to the culture medium. Before the pharmacological treatments, it was proven that the conditions of pollen germination and growth were optimal. After 30 min in the culture medium, about 70% of the pollens were germinated. After 1 h the germination rate was 90% and almost all the pollen tubes reached an average length of 230 μm, about seven times the diameter of the pollen (Fig. 1). The addition of the culture medium of BFA (90 min), IKA (100 min), and WMN (60 min) at a final concentration of 10 μM, 5 μM, and 30 μM respectively, have been shown to not be effective in altering pollen elongation. Similarity, the presence of only DMSO equivalent concentration had no effect on pollen tube elongation in respect to the control (only BK medium containing 12% sucrose). No solvent effect due to vesicle trafficking inhibitors or 0.2% DMSO, was evident comparing the morphology of treated and control pollen tubes (data not shown).

Since most of the purified proteins were soluble proteins, they were resuspended in the rehydration buffer and loaded onto linear pH 4–7 strips, being known that cytoplasmic proteins are relatively enriched in acidic residues such Asp, Glu [65]. In addition, 2D gel analysis using a pH interval of 3–10 showed that most of polypeptides were in the pH range of 4–7 (see supplementary material Fig. S1).

It seems that different isoelectric point (pI) values depend on protein subcellular localization. However, there is not always correlation between the protein's pI value and the optimum pH for its active form. For instance, concerning the cytoplasmic proteins they have an average pI ranging between 5 and 6, while the cytoplasmic pH is usually near neutrality; the acidic pI of cytoplasmic proteins could be required for their function in the cytosol. Vice versa, integral membrane proteins are enriched in nonpolar basic residues with pI around 9, in agreement with the negatively charged surface of the biomembranes [65].

**Fig. 2:**
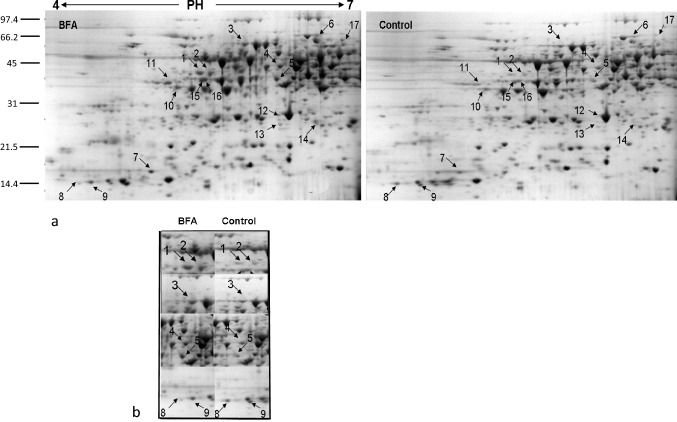
Pollen tube soluble proteome after BFA treatment. **a** 2-DE of *Nicotiana tabacum* pollen tubes after BFA treatment and related control. The DAPs are indicated by arrows and numbers corresponding to the spot ID in Table 1. **b** Magnified sections of the gels showing downregulation and upregulation of identified proteins after BFA. Spots are annotated as in panel a

**Fig. 3:**
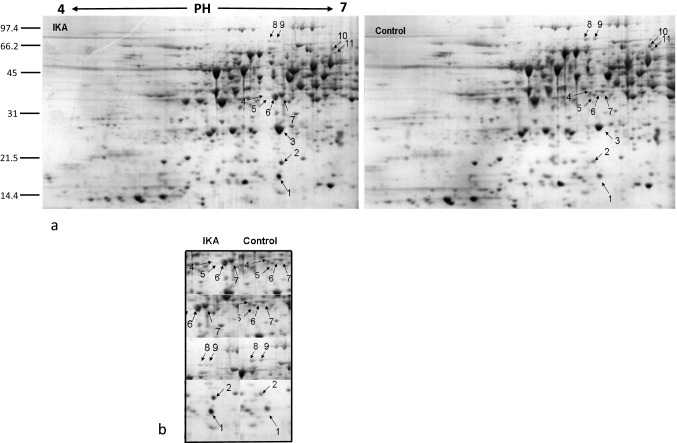
Pollen tube soluble proteome after IKA treatment. **a** Representative 2-DE of *Nicotiana tabacum* pollen tubes after IKA treatment and related control. Numbers correspond to the spot ID in Table 2. **b** Magnified sections of the gels showing downregulation and upregulation of identified proteins after IKA. Spots are annotated as in panel a

**Fig. 4:**
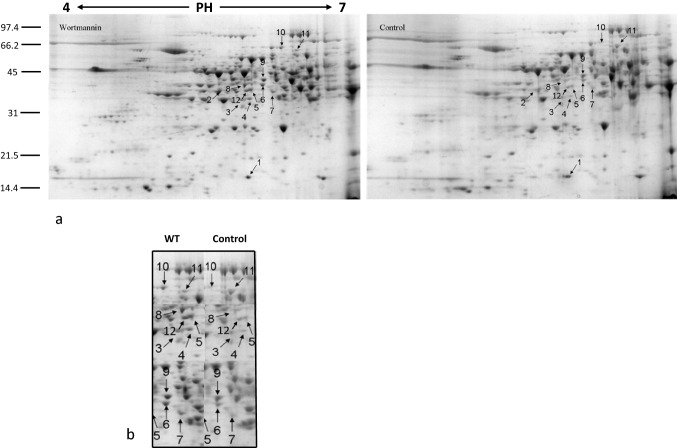
Pollen tube soluble proteome after WMN treatment **a** 2-DE of *Nicotiana tabacum* pollen tubes after WMN treatment and control. The DAPs are indicated by arrows and numbers corresponding to the spot ID in Table 3. **b** Magnified sections of the gels. Spots are annotated as in panel a

Following Coomassie staining, approximately 500 reproducible protein spots were detected per sample (Figs. 2a, 3a and 4a). All samples were analyzed in triplicates. Most spots were around pI 5–7, with molecular weights in the range 14–100 KDa. To determine DAPs between control and treated samples ANOVA statistical analysis was performed. All spots were matched by gel-to-gel comparisons and only proteins showing a statistically significant quantitative difference (p ≤ 0.05) were selected and manually picked up for digestion and identification by MALDI-TOF–MS analysis.

The ANOVA test, coupled with a threshold of 1.5-fold changes in levels, clearly revealed a total of 40 protein spots changing visibly (up- or down- regulated proteins) between treated and control samples as shown in Figs. 2a, 3a and 4a.

The excised spots were in-gel digested and analysed by MALDI-TOF mass spectrometry; 35 protein spots were successfully identified, corresponding to 22 unique proteins (Tables 1, 2 and 3). The experimental molecular masses (MW) and pIs of the majority of the identified proteins were consistent with the theoretical values, as judged by the location of the spots on 2-DE gels.

**Table 1:** Differentially abundant proteins after pollen tube treatment with BFA

Spot ID	Protein description	Accession number	Database	Mascot search results			Organism
				No. of matched peptides	Sequence coverage%	Score	
*Cytoskeletal proteins*							
1	Actin-54	ACT3_TOBAC	UniProtKB/Swiss-Prot	13/26	46	149	*N. tabacum*
2	Actin-104	ACT7_TOBAC	UniProtKB/Swiss-Prot	9/16	39	110	*N. tabacum*
8	Profilin	gi|6,469,497	NCBInr	5/5	39	88	*N. tabacum*
9	Profilin-3	gi|14,423,866	NCBInr	5/6	39	81	*N. tabacum*
*Energetic Metabolism*							
3	2,3-bisphosphoglycerate-independent phosphoglycerate mutase	PMGI_TOBAC	UniProtKB/Swiss-Prot	10/18	24	112	*N. tabacum*
6	Phosphoglucomutase cytoplasmic	PGMC_SOLTU	UniProtKB/Swiss-Prot	12/22	26	135	*S. tuberosum*
17	Phosphoglucomutase cytoplasmic	PGMC_SOLTU	UniProtKB/Swiss-Prot	7/16	17	71	*S. tuberosum*
10	Fructokinase	SCRK_SOLTU	UniProtKB/Swiss-Prot	12/18	37	127	*S. tuberosum*
*Lipid Metabolism*							
4	S-adenosylmethionine synthase 2	METK2_SOLTU	UniProtKB/Swiss-Prot	15/25	41	175	*S. tuberosum*
5	Glutamine synthetase OS	GLNA-NICPL	UniProtKB/Swiss-Prot	8/14	29	83	*N. plumbaginifolia*
11	Adenosine kinase isoform 2 T	gi|51,949,804	NCBInr	11/18	37 140	140	*N. tabacum*
*Glycosylation and polysaccharide biosynthesis*							
16	Alpha-1,4-glucan-protein synthase 2	UPTG2_SOLTU	UniProtKB/Swiss-Prot	9/12	17	71	*S. tuberosum*
15	Alpha-1,4-glucan-protein synthase 1	UPTG_MAIZE	UniProtKB/Swiss-Prot	GTLFPMCGMNLAFDR and ASNPFVNLK			*Z. mays*
*Protein degradation*							
12	Proteasome subunit alpha type-3	PSA3_ARATH	UniProtKB/Swiss-Prot	VPDDLLEEAK and AVDNSGTVVGIK			*A. thaliana*
	Proteasome subunit alpha type-3	PSA3_SPIOL	UniProtKB/Swiss-Prot	HSGMAVAGLAADGR			*S. oleracea*
13	Putative beta6 proteasome subunit	gi|14,594,933	NCBInr	DAVTPLSESEAIDLVK and SPSPLLLPAK			*N. tabacum*
Others							
7	Glycin-rich RNA-binding protein	gi|187,373,099	NCBInr	11/18	52	150	*N. tabacum*
14	Unidentified protein

**Table 2:** Differentially abundant proteins in tobacco pollen tubes after IKA treatment

Spot ID	Protein description	Accession number	Database	Mascot search results			Organism
				No. of matched peptides	Sequence coverage %	Score	
*Stress response proteins*							
*1*	Cytosolic class II small heat shock protein 4	gi|37,704,449	NCBInr	PNSYVFVVDMPGLK			*N. tabacum*
	Class II heat shock protein	HSP21_SOLPE	UniProtKB/Swiss-Prot	EYPNSYVFVVDMPGLK			*S. peruvianum*
	Class II heat shock protein	HSP18_ORYSJ	UniProtKB/Swiss-Prot	AMAATPADVK			*O. sativa subsp. japonica*
*Energetic Metabolism*							
3	Triosephosphate isomerase cytosolic isoform-like	gi|1,351,279	NCBInr	VASPAQAQEVHAELRK and WLQANVSAEVAASTR			*S. tuberosum*
4	Malate dehydrogenase^(a)^	A0A075F1V0_TOBAC	NCBInr	ANLGDEEIEALTK			*N. tabacum*
	Malate dehydrogenase^(a)^	MDHM_CAPAA	UniProtKB/Swiss-Prot	TQDGGTEVVEAK			*C. annuum var. annuum*
6	Malate dehydrogenase precursor	gi|2,827,080	NCBInr	6/16	20	54	*M. sativa*
7	Malate dehydrogenase	gi|10,798,652	NCBInr	14/30	55	134	*N. tabacum*
8	Plastid transketolase	gi|194,396,261	NCBInr	10/19	23	105	*N. tabacum*
9	Plastid transketolase	gi|194,396,261	NCBInr	9/11	17	117	*N. tabacum*
*Amino-acid biosynthesis*							
*4*	4-hydroxy-tetrahydrodipicolinate synthase*	DAPA_TOBAC	UniProtKB/Swiss-Prot	LPYVPLTK			*N. tabacum*
	4-hydroxy-tetrahydrodipicolinate synthase*	M1CQK5_SOLTU	UniProtKB/Swiss-Prot	VIGNTGSNSTR			*S. tuberosum*
*Others*							
11	T-complex protein 1 subunit eta	TCPH_ARATH	UniProtKB/Swiss-Prot	QLCDNAGFDATDVLNK and TFSYAGFEQQPK			*A. thaliana*
2	Eukaryotic translation initiation factor 5A-3	IF5A3_SOLTU	UniProtKB/Swiss-Prot	5/7	40	94	*S. tuberosum*
5	Unidentified protein						
10	Unidentified protein						

^(a)^The proteins identified by MALDI-TOF tandem MS confirmed the presence of two overlapping spots in the gel. Performing BLAST analysis between the matched peptides and the aminoacidic sequences of Malate dehydrogenase (A0A075F1V0_TOBAC and MDHM_CAPAA) and 4-hydroxy-tetrahydrodipicolinate synthase (DAPA_TOBAC and M1CQK5_SOLTU), the percentage of sequence identity is 100%

**Table 3:** Differentially abundant proteins after pollen tube treatment with WMN

Spot ID	Protein description	Accession number	Database	Mascot search results			Organism
				No. of matched peptides	Sequence coverage %	Score	
*Lipid Metabolism*							
9	S-adenosylmethionine synthase 2	METK2_SOLTU	UniProtKB/Swiss-Prot	9/13	34	133	*S. tuberosum*
1	Superoxide dismutase [Cu–Zn]	SODC_NICPL	UniProtKB/Swiss-Prot	6/7	50	121	*N. plumbaginifolia*
2	Glutamine synthetase	GLNA_NICPL	UniProtKB/Swiss-Prot	9/24	30	71	*N. plumbaginifolia*
5	Stearoyl-[acyl-carrier-protein] 9-desaturase, chloroplastic	STAD_SOLCO	UniProtKB/Swiss-Prot	8/23	28	71	*S. commersonii*
12	Stearoyl-[acyl-carrier-protein] 9-desaturase, chloroplastic	STAD_SOLCO	UniProtKB/Swiss-Prot	8/22	29	82	*S. commersonii*
*Energetic Metabolism*							
6	Phosphoglycerate kinase, cytosolic	PGKY_TOBAC	UniProtKB/Swiss-Prot	10/24	50	124	*N. tabacum*
8	Phosphoglycerate kinase, cytosolic	PGKY_TOBAC	UniProtKB/Swiss-Prot	10/16	42	142	*N. tabacum*
10	Plastid transketolase	gi|194,396,261	NCBInr	15/30	31	145	*N. tabacum*
*Cytoskeletal proteins*							
7	Actin	ACT3_TOBAC	UniProtKB/Swiss-Prot	7/28	35	62	*N. tabacum*
*Cysteine Metabolism*							
3	Putative cytosolic cysteine synthase 7	gi|76,556,494	NCBInr	13/25	53	158	*N. tabacum*
*Others*							
11 4	Unidentified protein Unidentified protein						

### The Effects of BFA on Pollen Tube Proteome

**Fig. 5:**
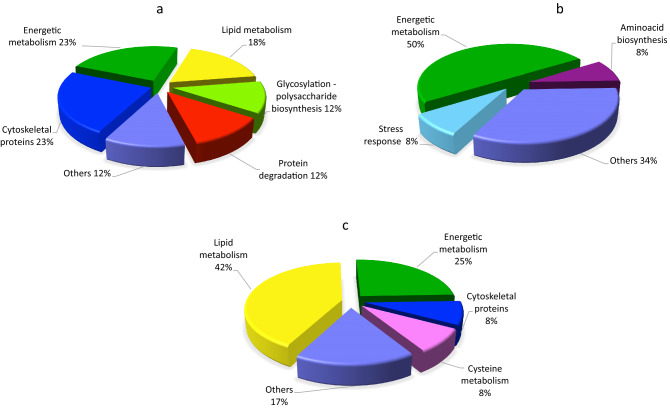
Classification of the identified proteins into functional groups. The pie charts show percentage distribution of the DAPs after BFA **a**, IKA **b** and WT **c** treatments, respectively

The inhibitor BFA, a macrocyclic lactone of fungal origin, is a powerful agent that has been extensively used in eukaryotic cells to prove vesicular trafficking pathways between the endoplasmic reticulum (ER) and the Golgi apparatus [57]. Previous studies indicate that BFA interferes in the formation of the Golgi associated coating proteins surrounding the “Coat Protein Complex I (COPI)-coated vesicles”, that are most likely involved in retrograde transport to the Golgi stacks [55]. In addition, BFA meddles the activity of GEFs (Guanine-nucleotide Exchange Factors) that catalyse the activation of ARF (ADP Ribosylation Factor), small GTPases that regulate vesicular traffic [36]. BFA has been widely used to study exocytosis and endocytosis during pollen germination and pollen tube growth [76], and here the effect of BFA on pollen tube vesicular traffic was evaluated. BFA-treated samples revealed the greatest number of DAPs compared to the other endocytosis inhibitors, perhaps because BFA has multiple targets at the cellular level. The optimized protein extraction resulted in successful resolution of the proteome on two-dimensional gels (Fig. 2a). Sixteen of the seventeen spots that passed quantitative computational analysis with a p < 0.05 were successfully identified and are listed in Table 1. Considering the expression differences, on average 14 up-regulated spots and 2 down-regulated spots changed about twofold after BFA treatment in comparison to the control. Samples were analysed in triplicates and all the replicates showed the same protein profiles, indicating subtle physiological changes due to the treatment rather than experimental errors. The number of peptides matched in the Mascot search program, the sequence coverage percentage and the score values are reported in Table 1. The relatively low number of proteins is mainly due to the applied ANOVA test *p*-value cutoff (0.05) for quantitative analysis. The proteins affected by BFA treatment were classified into functional groups (Fig. 5) and listed in Table 1.

#### Cytoskeletal Proteins

Numerous studies reported that filamentous actin play an important role in the internalization of PM vesicles [3]. On the other hand, disturbing pollen tube endomembrane trafficking with BFA causes the formation of a subapical Brefeldin A-induced membrane aggregation (BIA) and reorganization of the actin cytoskeleton [32], abolishing the F-actin structure at the apical region without affecting the longitudinal actin cables at the shank [83]. These results provided evidence that endomembrane trafficking and actin dynamics are conceivably dependent on each other. In agreement with literature, two actin isoforms in the soluble fraction (Fig. 2a and b, spots 1 and 2) resulted affected by BFA treatment (up-regulated), and that in presence of BFA actin binding proteins, such as profilin isoforms (Fig. 2a and b, spots 8 and 9) were down regulated. Profilin is known to be involved in actin cytoskeleton organization and turnover, and this data suggests that profilins modulate the actin cytoskeleton in a BFA dependent manner in *Nicotiana tabacum* growing pollen tubes. The actin binding proteins, such as profilin, are responsible for the actin dynamics in response to altered vesicular transport [70], so the differentially abundant profilin in BFA-treated pollen tubes compared to the control can be justified in response to altered vesicular and cytoskeletal processes.

Recent data revealed that formins AFH1 e AFH5 are involved in actin filament nucleation in the subapical and apical regions of tobacco pollen tubes, respectively ([15, 16]. In particular, it has been shown that AFH5 is associated with the apical plasma membrane matching the actin fringe. It is possible to hypothesize that BFA, disturbing membrane trafficking between ER and Golgi, inhibits the secretion of AFH5 to the plasma membrane, affecting the correct F-actin nucleation and inducing the BIA actin basket formation in the clear zone [32]. It is possible that an alteration of the actin filaments distribution/dynamic could increase the presence of actin which solubilizes during homogenization.

#### Energetic Metabolism

BFA induces the accumulation of three proteins involved in energy metabolism. The 2,3-bisphosphoglycerate-independent phosphoglycerate mutase involved in the glycolysis pathway, the phosphoglucomutase, an enzyme that participates in both the breakdown and synthesis of glucose and the fructokinase protein, involved in the pathway of starch biosynthesis, which is part of glycan biosynthesis. The increased protein levels (Fig. 2a, spots 3, 6, 17, and 10 respectively) suggest that BFA treatment could affect the intracellular levels of the enzymes. An accumulation of the enzymes involved in energy metabolism could be related to possible interference in energy-consuming processes, such as actin polymerization and exo- and endocytosis, following BFA treatment.

#### Lipid Metabolism

The accumulation of proteins involved in fatty acid synthesis and metabolism have been detected: S-Adenosyl methionine synthase 2, Glutamine synthetase and Adenosine kinase (ADK) (Fig. 2a and b, spots 4, 5, and 11 respectively). The S-Adenosyl methionine synthase (SAMS) together with the methionine synthase (MetE), are enzymes involved in the synthesis and regeneration of S-adenosylmethionine (SAM). SAMS, also known as methionine adenosyltransferase (MAT), is an enzyme that catalyzes the formation of SAM by the transfer of adenosine (Ado) from ATP to methionine [78]. SAM is a cofactor required as a methyl donor in the biological methylation reactions, and in eukaryotes participates in numerous enzyme-catalysed reactions including lipid metabolism [72]. The enzymes that control SAM levels play a critical role in determining the extent of methylation. MetE has been identified in tobacco pollen tubes, on the surface of vesicles accumulated in the clear zone where also sterol-rich vesicles have been localized [45, 47]. Moreover, MAT has been revealed as detergent insoluble microdomains (DIMs)-enriched protein suggesting that DIMs could be sites for S- methylmethionine cycle [45]. Recently, Chen et al. [14] showed that the MAT3 gene is highly expressed in pollen and that a *mat3* knockdown mutant shows an higher content of fatty acids than the wild type. The alteration of SAMS and ADK involved in maintaining transmethylation activity [42] following BFA treatment, may contribute in explaining the importance of particular metabolic pathways during pollen tube growth.

BFA brings about a Glutamine synthetase accumulation, an enzyme that assimilates ammonium into aminoacids. The cytosolic isoform of Glutamine synthetase is particularly important for assimilating ammonium from different sources, for both primary nitrogen assimilation and recycling [4]. It has been reported in diatom and microalgae that lower nitrogen concentration favored a higher lipid content percentage compared to higher nitrogen concentrations [80, 81], suggesting an effect of nitrogen on growth, lipid production and fatty acid profiles. Thus, a role of Glutamine synthetase in lipid metabolism could also be supposed in pollen tubes, with a possible involvement in the membrane lipid synthesis and in the production of exocytotic vesicles delivered to the pollen tube apex for their fusion with the PM.

#### Glycosylation and Polysacchrides Biosynthesis

Alpha-1,4-glucan-protein synthase belongs to the glucosyltrasferase protein family and is involved in plant emicellulose polysaccharides biosynthesis [74]. Recent studies showed that this enzyme plays a key role in quality control mechanism of newly synthesized glycoproteins in the endoplasmic reticulum [6], suggesting that it is a secretory pathway resident protein. In addition the presence of α 1,4 glucan protein synthase in the soluble fraction suggests that endomembrane resident proteins could also be solubilized during the homogenization and HSS preparation. Membrane trafficking is a key mechanism for transporting many proteins to the plasma membrane or extracellular space to properly generate and modify the cell wall. Generally, the transport vesicles bud from donor membranes to the target membrane and could be mediated through coat protein complexes. The newly-formed vesicles are transported and tethered to the target membrane through GTPases and as final step, SNARE proteins execute membrane fusion [22]. Considering the ability of BFA to alter vesicular traffic and F-actin organization, it is possible to hypothesize its role in inhibiting the turnover of enzymes associated with the organization of the cell wall.

#### Protein Degradation

Spots corresponding to proteolytic enzymes, such as proteasome subunit alpha-type-3 and beta-6 (Fig. 2a, spots 12 and 13 respectively) were up-regulated suggesting that proteolytic processes might be altered by BFA, as already reported by Takàc et al. [70]. More deepened studies will be necessary to understand the causes of this proteasome increase, that could be linked to various effects including the possible accumulation of proteins in the ER caused by e ER-Golgi transport inhibition following treatment with BFA. It seems that degradation by the ubiquitin/proteasome system (UPS) also plays a major role in regulating the level of proteins synthesized within the ER. Indeed, the ER associated degradation (ERAD) functions as a quality control machinery [71].

### IKA Affects Abundance of Pollen Tube Proteins

IKA is an antibiotic which has been used to inhibit clathrin-mediated endocytosis (CME) in plant cell [44, 51]. The function of this drug is similar to that of Tyrphostin [52] but IKA is less toxic for pollen tubes growth and a 5 μM solution allows the pollen tubes to growth, although into a slower manner compared to the control.

The use of positively and negatively charged nanogold in combination with IKA allows to dissect the endocytic pathway in growing pollen tubes [30, 44]. In particular, it has been shown that IKA affects internalization in the apical and subapical regions of pollen tubes, leading to the partial inhibition of the PM recycling into the Golgi/secretory pathway. Electron microscopy showed that Golgi bodies were stained by positively charged nanogold in the presence of IKA, whereas the degradative pathway did not seem to be affected, suggesting that clathrin-dependent and -independent endocytosis both occur in pollen tubes [44, 51]. After IKA treatment, PDQuest analysis revealed eleven spots that passed the p-value filter (p < 0.05) to be considered differentially abundant. Nine spots, successfully identified are listed in Table 2. On average 5 up-regulated spots and 6 down-regulated spots changed about twofold after IKA treatment in comparison to the control. Like BFA treatment, the proteins affected by IKA were classified into functional groups (Fig. 5). The protein identified as differentially abundant are listed in Table 2.

#### Stress Response Proteins

IKA treatment causes a cytosolic class II small heat shock protein (Fig. 3a and b, spot 1) increase. In response to high temperatures and other abiotic stresses, all organisms respond by accumulating small heat shock proteins (sHSPs) [40]. The ubiquitous sHSPs act as molecular chaperones and are involved in various molecular mechanisms like the assembly and transport of proteins, the maintenance of the protein in a normal folding state, the protection against irreversible protein’s denaturation [48]. In tobacco pollen, cytosolic class I and II sHSP genes are expressed in a stage-specific fashion suggesting that different sHSP genes play specific roles in early and/or later stages of pollen development [73]. In our studies, the increased quantity of the cytosolic class II sHSPs in response to a stress condition caused by the IKA treatment, could be related to an incorrect assembly of clathrin into the clathrin-coated vesicles.

#### Energetic Metabolism

Three different proteins involved in the energetic metabolism have been affected by IKA treatment: the cytosolic isoform of triosephosphate isomerase (cTPI) (Fig. 3a, spot 3), the malate dehydrogenase and the malate dehydrogenase precursor (MDH) (Fig. 3a and b, spots 4, 7 and 6 respectively), and the plastid transketolase (TK) an enzyme involved in the Calvin Benson cycle with a significant effect on photosynthesis and growth in tobacco plants [37], (Fig. 3a and b, down-regulated 8 and 9 spots.

TPI is present in plants in both the cytosolic and plastidial forms, and it is involved in many pathways including glycolysis, the Calvin cycle, and glycerol metabolism [12]. It has also been shown that S-glutathionylation of cTPI in *A. thaliana* could be involved in the response to oxidizing conditions in the cytoplasmic compartment [21]. The up regulation of this enzyme in the presence of IKA could be linked to both the energetic metabolism alteration and the defence mechanisms against oxidative stress. Also, IKA is responsible for the changes in MDHs’ concentration. MDHs, already identified during pollen proteome analyses of different plant species [68, 86], catalyse the interconversion of malate into oxaloacetate using the reduction of NAD^+^ to NADH [34]. MDHs play important roles in plant growth and development, and several isoforms have been identified differing in their subcellular localization, malate selectivity and reaction requirements [34]. Pollen tube growth occurs in a very fast way and it is an high energy-consuming process. Different energy-generating pathways are active in pollen tubes as mitochondrial respiration, fermentation and plastidial glycolysis, contributing to satisfy the energy demand during pollen tube development [66]. MDHs isoforms are located in the mitochondria, cytosol, peroxisomes and plastids, although mitochondrial and cytosolic ones are the most abundant [82]. It has been shown that as component of malate valves, MDHs play a key role in energy homeostasis, while plastid NAD-MDHs are responsible for the NAD^+^ regeneration to maintain ATP production by plastidial glycolysis, and the mitochondrial MDH isoforms seem to be involved of NADH regeneration used in respiration process [67]. The increase of MDHs in the pollen tubes after treatment with IKA could be related to an alteration of energy-consuming processes like endocytosis and the actin cytoskeleton stability [15].

The up/down regulation of enzymes involved in cellular energy metabolism supports that pollen tube development is engaged in a very high metabolic activity, probably partly inhibited by the presence of IKA. Indeed, the regulation of energy metabolism during pollen tube growth is very complex and deepened studies are still necessary.

#### Amino-acid Biosynthesis

Spot 4 (Fig. 3a and b), includes two different proteins, a putative mitochondrial MDH enzyme isoform (as above described) and the cloroplastic 4-hydroxy-tetrahydrodipicolinate synthase (DHDPS, DapA), a homotetrameric enzyme of lysine biosynthesis, catalyzing the condensation of (S)-aspartate-beta-semialdehyde [(S)-ASA] and pyruvate to 4-hydroxy-tetrahydrodipicolinate (HTPA). Lysine biosynthesis is related to arabinogalactan proteins (AGPs) in pollen. AGPs are complex proteoglycans involved in pollen tube growth guidance and in the fertilization process [50]. Zhang et al. [85] reported that *At*AGP18, a lysine-rich AGP, would seem to work as a putative co-receptor for signal transduction to control plant growth. In this model, *At*AGP18 located on the outer surface of the plasma membrane in lipid rafts could act as a co-receptor to take over extracellular signals and interact with transmembrane proteins to initiate signaling by starting various intracellular events.

### WMN Influences the Soluble Proteome

WMN is a fungal steroid metabolite utilized to disturb endomembrane trafficking by the inhibition of protein vacuolar sorting and endocytosis [83]. WMN specifically inhibits Phosphatidylinositol 3-Kinase (PI3K) but at higher concentrations it can affect both PI3K and Phosphatidylinositol 4-Kinase (PI4K) [69]. Phosphatidylinositol (PI) is involved in stress or developmental signals and can modulate vesicular trafficking, stress response and cell/organ development. PI3K and PI4K are responsible for the production of Phosphatidylinositol 3 phosphate (PI3P) and phosphatidylinositol 4 phosphate (PI4P) the principal components of PI signaling. PI3P and PI4P recruit PI3P and PI4P-binding proteins to the membranes and mediate membrane fusion and trafficking in the cell [69].

On the proteome level, WMN-treated tobacco pollen tubes show changes in protein abundance profiles. Twelve spots have been considered differentially abundant passing the p-value filter (p < 0.05). Ten spots successfully identified are listed in Table 3. WMN-sensitive proteins belong to various functional classes (Fig. 5).

#### Lipid Metabolism

The proteins related to lipid metabolism represent the prevalent functional group (Fig. 5). Similar to BFA, WMN treatment affect S-Adenosyl methionine synthase 2 (SAMS-2) and Glutamine synthetase (Fig. 4a, spots 9 and 2, respectively), protein that seem to be involved in lipid content regulation. More, the presence of WMN in the growth medium causes an accumulation of two other proteins, superoxide dismutase (SOD) and Stearoyl-[acyl-carrier-protein] 9-desaturase (SAD) (Fig. 4a and b, spots 1, and 5 and 12, respectively). Within a plant cell, SODs destroy radicals which are produced within the cells and which are toxic to biological systems. SODs constitute the first line of defence in controlling oxidative stress, including lipid peroxidation [1]. In plant cells, biotic and abiotic stress or tissue senescence can increase the production of active oxygen species (AOS) causing cellular damage such as the oxidation of phospholipids and other unsaturated lipids. Peroxidation results in the breakdown of lipids and membrane function by causing loss of fluidity, lipid crosslinking, and inactivation of membrane enzymes [18]. Finally, SAD is involved in the metabolic pathway of fatty acids. SAD’s activity has significant effects on the ratio of saturated fatty acids to unsaturated fatty acids, and as a consequence it has an important effect on the fluidity and rigidity of membrane system [84]. The up-regulation of this protein after WMN treatment recalls the action of this metabolite in disturbing cellular endomembrane trafficking.

#### Cytoskeletal Protein and Energetic Metabolism

WMN treatment increases the abundance of actin in pollen tubes (Fig. 4a and b, spot 7), similarly to BFA. WMN causes changes in proteins involved in the energetic metabolism like the plastid TK protein (Fig. 4a and b, spot 10). The plastid TK protein, involved in the Calvin cycle, seems to be up-regulated by WMN and down-regulated by IKA.

The up-regulated spots 6 and 8 (Fig. 4A) refer to two isoforms of the cytosolic phosphoglycerate kinase (PGKc), a protein involved in glycolysis. Chen et al. [13] identified a PGKc-1 mutant with compromised pollen tube growth polarity and reported the glycolysis pathway plays a new and unconventional role in the regulation of cell polarity in *Arabidopsis* pollen tubes. The PGKc-1 mutation is associated with an over-activation of Rho GTPase, and consequently PGKc-1 pollen tubes contain higher amounts of exocytic vesicles and actin microfilaments in the apical region. After WMN treatment, the up-regulation of PGKc and actin in tobacco pollen tubes could be associated with a disturbance in the establishment and maintenance of cell polarity.

#### Cysteine Metabolism

The plant cysteine synthase complex (CSC), (Fig. 4a and b, spot 3) is located not only in plastids but also in mitochondria and the cytosol [61, 62]. CSC, which is formed by association of serine acetyltransferase (SAT) and O-acetylserine (thiol) lyase (OAS-TL), is responsible for regulation of cysteine synthesis and forms a sensor system that is involved in the control of sulphur metabolism in plant cells [79]. Cysteine is the metabolic precursor of some biomolecules such as antioxidants and vitamins [2]. Cytosolic cysteine plays an important role in plant immunity, while in the mitochondria it is responsible in the detoxification of cyanide, a process necessary for root hair development [58]. Furthermore, small cysteine-rich proteins (CRPs) have been found to play multiple roles in plant reproduction. Recent studies [29] report that as in *Arabidopsis*, receptor kinases (PRKs) interact with a guanine nucleotide exchange factor (RopGEF) and are able to directly activate a pollen-specific ROP GTPase, playing a central role in determining the polarity of the pollen tube at the apical plasma membrane. It has been shown that as PRKs are able to interact with a variety of ligands, including a cysteine-rich extracellular protein (LAT52), which is essential in mediating reproductive success.

## Conclusions

Pollen tube growth depends on complex signaling pathways, involving Rho GTPases and their effectors which, in turn regulate actin dynamics in the tip and the polarized exocytosis of Golgi derived secretory vesicles [25, 38]. Recent studies revealed that distinct clathrin-dependent and clathrin-independent endocytic pathways are involved in retrieving the excess of secreted plasma membrane that could be addressed to recycling or to degradation [44]. While it is known that secretion, clathrin-dependent endocytosis and late endosome-vacuole fusion are highly integrated processes and are inhibited by the use of BFA, IKA and WMN, respectively, the aim of our approach was to investigate any effects of the inhibitors on protein abundance.

Intriguingly, our results showed that the use of the drugs has a pleiotropic effect on pollen tube proteome, by altering not only proteins involved in membrane trafficking, but also proteins linked to energy metabolism and structural molecules.

**Fig. 6:**
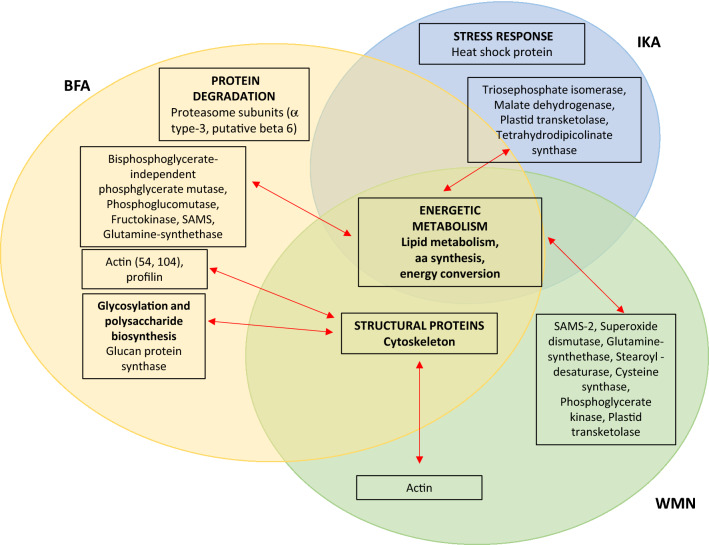
Overview on BFA, IKA and WMN interference on biochemical pathways of *N. tabacum* pollen tube. BFA, IKA and WMN act on the energetic metabolism (lipid metabolism, amminoacid-synthesis, and energy conversion); BFA and WMN act on main structural features of the cell, such as the cell wall and cytoskeleton; activation of the protein degradation pathway and the stress response seem to be elicited, respectively by BFA and IKA. Even when sharing common metabolic targeted pathways, each drug seems to produce specific biochemical changes in the *N. tabacum* pollen tube proteomics profiling

After drug treatments, the DAPs in pollen tubes represent about 10% of the total proteins expressed in the controls. Many of the targeted proteins are involved in energetic metabolism. Pollen tube growth is a complex mechanism strongly dependent on energy-consuming processes such as exo- and endocytosis, or actin polymerization that can be affected by the drugs (Fig. 6). Other proteins highly influenced by treatment of the pollen tubes with inhibitors of vesicular traffic are those involved in lipid metabolism and cytoskeletal organization. The identification of different actin isoforms and proteins involved in lipid synthesis suggests that pollen tubes polarized tip growth could require complex molecular mechanisms integrating both the secretory pathway and the dynamic of actin cytoskeleton, which in turn seems to be regulated by lipid signaling [26].

Moreover, the data obtained by the proteomic analysis are functional to deepening the physiology of pollen tube growth regulation especially considering that recent gene expression studies are focused on RNAseq or massive analysis through on gene chip transcriptomics. As reported by Fíla et al. [24], for systems with high level of translational regulation, such as the male gametophyte the transcriptional analysis must be validated by proteomic studies. As a matter of fact, translation and mRNA storage are active mechanisms during male gametophyte development and it is desirable to complement transcriptomics with proteomic data to get more realistic insights.

## Supplementary Information

Below is the link to the electronic supplementary material.

Fig. S1 Soluble proteome of Nicotiana tabacum pollen tubes, grown up without membrane trafficking inhibitors. 2D gel analysis using a pH interval of 3-10 shows that most of polypeptides are in the pH range of 4-7 (PPTX 104 KB)

## Data Availability

The authors confirm that the data supporting the findings of this study are available within the article and its supplementary materials.
