# Supplementary figures and images for: Protein Analysis of Pollen Tubes after the Treatments of Membrane Trafficking Inhibitors Gains Insights on Molecular Mechanism Underlying Pollen Tube Polar Growth
Source: Protein J. 2021 Mar 9;40(2):205–22. doi: 10.1007/s10930-021-09972-x (PMC8019430; doi:10.1007/s10930-021-09972-x)

## Slide 1
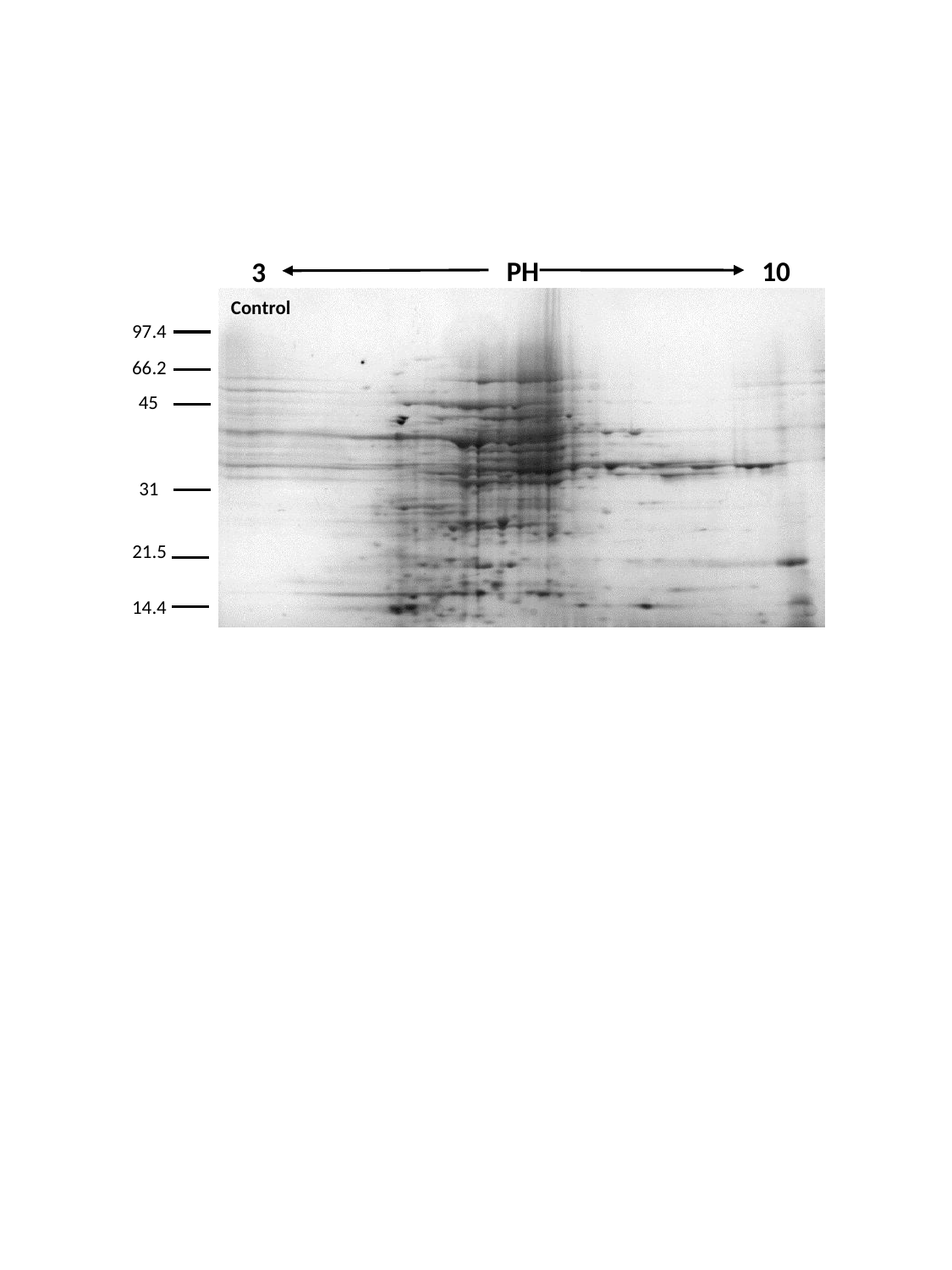

PH
10
3
Control
97.4
66.2
45
31
21.5
14.4

Supplement: Supplementary file 1 — Fig. S1 Soluble proteome of Nicotiana tabacum pollen tubes, grown up without membrane trafficking inhibitors. 2D gel analysis using a pH interval of 3-10 shows that most of polypeptides are in the pH range of 4-7 (PPTX 104 KB) [file 10930_2021_9972_MOESM1_ESM.pptx]
